# Acute healthcare utilization in end-of-life among Swedish brain tumor patients – a population based register study

**DOI:** 10.1186/s12904-022-01022-2

**Published:** 2022-07-23

**Authors:** Magnus Lindskog, Torbjörn Schultz, Peter Strang

**Affiliations:** 1 Department of Immunology, Genetics and Pathology, Uppsala University, Uppsala, Sweden; 2 Department, Stockholms Sjukhem Foundation, Stockholm, Sweden; 3 Department of Oncology-Pathology, R & D Department, Karolinska Institutet, Regional Cancer Centre in Stockholm – Gotland, Stockholms Sjukhem Foundation, Stockholm, Sweden

**Keywords:** Brain tumor, Palliative care, Healthcare utilization, Hospital, Register

## Abstract

**Background:**

Patients with progressive primary brain tumors commonly develop a spectrum of physical as well as cognitive symptoms. This places a large burden on family members and the condition’s complexity often requires frequent health care contacts. We investigated potential associations between sociodemographic or socioeconomic factors, comorbidity or receipt of specialized palliative care (SPC) and acute healthcare utilization in the end-of-life (EOL) phase.

**Methods:**

A population-based retrospective study of all adult patients dying with a primary malignant brain tumor as main diagnosis in 2015–2019 in the Stockholm area, the most densely populated region in Sweden (*N* = 780). Registry data was collected from the Stockholm Region´s central data warehouse (VAL). Outcome variables included emergency room (ER) visits or hospitalizations in the last month of life, or death in acute hospitals. Possible explanatory variables included age, sex, living arrangements (residents in nursing homes versus all others), Charlson Comorbidity Index, socio-economic status (SES) measured by Mosaic groups, and receipt of SPC in the last three months of life. T-tests or Wilcoxon Rank Sum tests were used for comparisons of means of independent groups and Chi-square test for comparison of proportions. Associations were tested by univariable and multivariable logistic regressions calculating odds ratios (OR).

**Results:**

The proportion of patients receiving SPC increased gradually during the last year of life and was 77% in the last 3 months of life. Multivariable analyses showed SPC to be equal in relation to sex and SES, and inversely associated with age (*p* ≤ 0.01), comorbidity (*p* = 0.001), and nursing home residency (*p* < 0.0001). Unplanned ER visits (OR 0.41) and hospitalizations (OR 0.45) during the last month of life were significantly less common among patients receiving SPC, in multivariable analysis (*p* < 0.001). In accordance, hospital deaths were infrequent in patients receiving SPC (2%) as compared to one in every four patients without SPC (*p* < 0.0001). Patients with less comorbidity had lower acute healthcare utilization in the last month of life (OR 0.35 to 0.65), whereas age or SES was not significantly associated with acute care utilization. Female sex was associated with a lower likelihood of EOL hospitalization (OR 0.72). Nursing home residency was independently associated with a decreased likelihood of EOL acute healthcare utilization including fewer hospital deaths (OR 0.08–0.54).

**Conclusions:**

Receipt of SPC or nursing home residency was associated with lower acute health care utilization among brain tumor patients. Patients with more severe comorbidities were less likely to receive SPC and required excess acute healthcare in end-of-life and therefore constitute a particularly vulnerable group.

## Background

In Sweden, about 1300 persons are diagnosed with a brain tumor each year. The median survival for the most common high-grade primary brain tumor in adults, glioblastoma, is in the range of 16–21 months, with very few long-term survivors [1] and in fact, brain tumors constitute the fourth leading cause of years lost to cancer [2]. According to the Swedish National Cause of Death Register on average about 600 patients die annually with a malignant brain tumor as their underlying cause of death.

Already at diagnosis, many brain tumor patients have disturbing symptoms and might need support and palliative interventions. [3]. At the end-of-life (EOL), most of these symptoms are aggravated; typically including headache, nausea, seizures, fatigue, mood and behavioral disorders, reduction in cognitive abilities, and palsies [3–7]. This places a heavy burden on both patients and family members whom often become informal caregivers despite that their need for support is not always acknowledged [8, 9].

The palliative care needs during the EOL might be of a general nature, which can be provided in nursing homes or, in acute situations, at acute hospitals. However, most cancer patients, including those with brain tumors, tend to have complex symptoms and needs that are best handled by specialist palliative care (SPC) services.

In Sweden, SPC is mainly offered in the form of advanced palliative home care and, when needed, by hospital palliative care units. Both types of care are staffed around the clock, with physicians, registered nurses, physiotherapists, occupational therapists, dieticians, assistant nurses, and other medical professionals [10]. These services are highly appreciated, both by patients and their families [10].

In general terms, ASCO (American Society of Clinical Oncology) recommends early integration of palliative care into standard oncology care [11]. Still, this is not always the case for persons with brain tumors, in fact referrals to palliative or hospice care tend to be rather late, as reviewed by Wu et al. [1].

Previous studies indicate that, receipt of SPC, may decrease the need for unplanned emergency room (ER) visits, a care environment that is less than optimal for palliative care patients, especially in EOL situations [12]. In contrast, overly aggressive EOL care including repeated hospitalizations and ICU is often associated with suboptimal EOL care, according to family caregivers [13]. In a Swedish context, we previously reported an inverse association of hospital deaths and EOL care quality for cancer patients [14].

Several studies [15–17] have highlighted inequity in cancer care, early in the disease trajectory as well as in the EOL phase. Inequity is important to detect and counteract and equal care is a stated goal in the Swedish Cancer plan. Specifically, patients with advanced life-limiting disease and complex needs should be offered SPC and the referrals should not depend on aspects such as sociodemographic factors, socioeconomic status or comorbidity, [17–19].

Relatively little is known about the care received by brain tumor patients in their last year of life although some data exist. A case–control study found hospitalizations within a month of death to be frequent in glioblastoma patients [20]. A Canadian study likewise found the burden of care to be significant in the last 6 months of life for brain tumor patients, and comorbidity and age were associated with higher acute care utilization [21]. An Australian retrospective cohort study found receipt of palliative care to be inversely associated with hospital deaths among patients with malignant glioma [22].

In the present study, we have investigated potential associations between socioeconomic or sociodemographic factors, comorbidity or receipt of SPC and end-of-life acute care utilization among brain tumor patients in the context of a publicly funded healthcare system.

### Aims

To explore possible associations of patient- and care-related factors, including receipt of specialized palliative care (SPC) in the last three months of life, comorbidity, socioeconomic or sociodemographic factors, with acute healthcare utilization in the last month of life among brain tumor patients.

## Methods

The Methods and the Results sections are, when possible, reported based on the Strengthening the Reporting of Observational Studies in Epidemiology (STROBE) criteria [23].

### Study design

This was a retrospective observational registry data study based on the Stockholm Region´s central data warehouse (VAL) and data were collected for five consecutive years, 2015–2019. In the Stockholm region, all appointments, hospital visits, ICD-10 diagnoses, and major costs are registered by respective caregiver and stored in VAL. As reporting of data to VAL is mandatory for each clinic/care unit, data are complete and very few values are missing. Each person who has used public health care during the study years is included in the VAL database, which also includes most forms of private care, as private care providers have economic agreements with the regional council.

The Swedish healthcare system is tax funded and publicly available to all citizens, which enabled a population-based inclusion of study subjects.

### Population

All patients over the age of 18 years (childhood cancers were excluded), who had died during 2015–2019 with a main diagnosis of malignant neoplasms of brain (ICD-10 code C71) were included.

### Variables

ER visits, hospitalizations during the last month of life and hospital deaths were used as outcome measures. As explanatory variables, receipt of SPC, age, sex, living arrangements (nursing home residents versus all others), comorbidities in the form of Charlson Comorbidity Index (CCI), and socio-economic status by means of Mosaic were used. Mosaic is a system that provides socio-economic information based on the actual living area. Thus, the county of Stockholm is divided into 1300 small areas and each area is classified as Mosaic 1, 2 or 3, mainly based on income and education, but also, for example, on cultural aspects, lifestyle and living arrangements. The three groups are approximately equal in size. Mosaic group 1 corresponds to the most affluent areas [24–26]. For each patient, data on living arrangements and Mosaic groups were based on the last known address. The CCI is a method of categorizing comorbidities of patients based on the ICD-10 diagnosis codes found in administrative data and is often used as a proxy for comorbidity burden [27]. The lookback window was 3 years from the time of death for each patient. As all included patients had cancer, this variable was excluded from the CCI index.

### Selection bias

**Dropouts**: As reporting of data to the administrative databases is mandatory for each clinic/care unit, and also basis for each clinic´s economic compensation, data is complete and very few values are missing.

**Nursing home residents**: To identify nursing home residents, registrations of medical interventions by doctors were identified. There are exclusive codes for physician care in nursing homes.

**The Charlson Comorbidity Index:** As relevant diagnoses are registered for each outpatient visit and every hospitalization, comorbidities relevant to the index have few missing values.

**Mosaic**: As Mosaic is based on a permanent or temporary living address. As a few individuals have no address, Mosaic cannot be assigned for these persons.

**Missing data:** There were missing registrations in MOSAIC for 12 individuals. These were excluded from the regression analyses.

### Study size

The study comprised the whole cohort for the years 2015–2019. Therefore, no power calculation was made.

### Statistical methods

T-tests were used for age, Wilcoxon Rank Sum test (Mann Whitney U test) were used for comparisons with skew distributions and Chi-square tests for comparison of proportions. Initially, univariable logistic regression analyses were performed for relevant variables, which then were entered into fully adjusted logistic regression models. The SAS 9.4/Enterprise guide 8.2 was used for statistics.

### Ethics

The study was approved by the Regional Ethical Review Authority (EPN 2017/1141–31).

## Results

### Study subjects

We identified 780 patients who died with a malignant brain tumor as the main diagnosis during the study period of 2015 – 2019. Table 1 shows the overall characteristics of the cohort. There was a slight male predominance but sex was not associated receipt of SPC, nursing home residency during the last year of life, socio-economic Mosaic groups or comorbidity. Female patients were somewhat older, 66.9 years versus 63.8 years (*p* = 0.002; data not shown in tables). The mean modified Charlson Comorbidity Index (CCI) was 0.90 (range 0–7), 91% had values between 0–2.

**Table 1:** Characteristics and care utilization for 780 patients who died with a malignant brain tumors as main diagnosis

Characteristics	Total (*n* = 780)
**Age**, mean years (sd)	65.1 (14.1)
**Mosaic**	
Group 1, n (%)	238 (31)
Group 2, n (%)	329 (42)
Group 3, n (%)	213 (27)
**CCI**	
0 – 1, n (%)	383 (49)
> 1, n (%)	397 (51)
**SPC** (%) ^*^, n (%)	604 (77)
**Care in nursing homes,** n (%)	160 (21)
Age in nursing homes, mean years	72.4

MOSAIC groups = socioeconomic entities based on small geographical areas, Mosaic group 1 corresponding to the most affluent one; *SPC* specialized palliative care, *CCI* Charlson comorbidity index

^*^ Patients receiving SPC in the last three months of life

### Access to specialized palliative care in the last three months of life

The proportion of patients receiving specialized palliative care (SPC) increased gradually during the four quarters of the last year of life as follows: 6%—10%—22%—77%. In univariable analyses, the proportion of patients with SPC during the last three months of life was associated with younger age and lower CCI indices, and inversely with being a nursing home resident (Table 2). These three variables also remained significant in multivariable analyses, although the association with age was weakened in the fully adjusted model. Sex was not associated with receipt of SPC. Patients belonging to higher socio-economic Mosaic groups were somewhat more likely to receive SPC, in univariable comparisons, but the association was weakened in the full multivariable model.

**Table 2:** Factors associated with receipt of SPC during the last 3 months of life among brain tumor patients

Variable		Univariable analysis		Multivariable analysis	
	*N* = 780	OR (95% CI)	*p*-value	OR (95% CI)	*p*-value
Sex					
Women	328	0.86 (0.61 – 1.21)	0.39	1,02 (0.69 – 1.52)	0.90
Men	452	Ref		Ref	
Socio-economic status					
Mosaic group 1	238	1.30 (0.92 – 2.14)	0.12	1.49 (0.90 – 2.45)	0.12
Mosaic group 2	329	1.84 (1.22 – 2.77)	0.003	1.73 (1.08 – 2.77)	0.02
Mosaic group 3	213	Ref		Ref	
Age groups					
18 – 64 years	327	7.88 (4.00 – 15.52)	< 0.0001	3.03 (1.37 – 6.70)	0.006
65 – 74 years	245	4.38 (2.23 – 8.58)	< 0.0001	2.70 (1.23 – 5.93)	0.01
75 – 84 years	165	3.94 (1.96 – 7.92)	0.0001	2.50 (1.11 – 5.66)	0.03
85 years or older	43	Ref		Ref	
Comorbidity (CC1)					
0 – 1	383	2.22 (1.57 – 3.16)	< 0.0001	1.90 (1.27 – 2.84)	0.002
> 1	397	Ref		Ref,	
Nursing home resident					
yes	160	0.10 (0.06 – 0.14)	< 0.0001	0.11 (0.07 – 0.16)	< 0.0001
no	660	Ref		Ref	

*OR* Odds ratio, *CCI* Charlson comorbidity index, MOSAIC groups = socioeconomic entities based on small geographical areas, Mosaic group 1 corresponding to the most affluent one

### ER visits during the last month of life

In total, 213/780 (27%) of the patients made unplanned ER visits, significantly less for those receiving SPC compared to others: 24% versus 39% (*p* < 0.0001) (Table 3). In logistic regression models, especially receipt of SPC but also lower CCI indices were inversely associated with ER visits, both in univariable and multivariable models (Table 4). Nursing home residency was a non-significant variable in univariable analysis but became strongly significant (inverse association) in the fully adjusted model with an OR of 0.46 (0.28 – 0.76), *p* = 0.002 (Table 4).

**Table 3:** Acute healthcare utilization during the last month of life among brain tumor patients in relation to receipt of specialized palliative care

Care utilization	Total	With SPC	Without SPC	*p*-value ^1^
Emergency room visits	213/780 (27%)	144/604 (24%)	69/176 (39%)	< 0.0001
Hospital admissions	258/780 (33%)	181/604 (30%)	77/176 (44%)	0.0006
Hospital as place of death	60/780 (8%)	14/604 (2%)	46/176 (26%)	< 0.0001

^1^Chi-2; SPC = receipt of specialized palliative care in the last three months of life

**Table 4:** Emergency room visits during the last month of life among brain tumor patients

Variable	Univariable analysis		Multivariable analysis	
	OR (95% CI)	*p*-value	OR (95% CI)	*p*-value
Sex				
Women	0.86 (0.62 – 1.19)	0.36	0.83 (0.59 – 1.15)	0.26
Men	Ref		Ref	
Socio-economic status				
Mosaic group 1	0.63 (0.42 – 0.95)	0.03	0.68 (0.44 – 1.04)	0.34
Mosaic group 2	0.73 (0.50 – 1.77)	0.11	0.80 (0.54 – 1.19)	0.20
Mosaic group 3	Ref		Ref	
Age groups				
18 – 64 years	0.49 (0.25 – 0.94)	0.03	0.55 (0.27 – 1.13)	0.10
65 – 74 years	0.57 (0.29 – 1.13)	0.11	0.66 (0.32 – 1.35)	0.26
75 – 84 years	0.66 (0.33 – 1.33)	0.66	0.72 (0.34 – 1.50)	0.38
85 years or older	Ref		Ref	
Comorbidity (CC1)				
0 – 1	0.72 (0.56 – 0.93)	0.01	0.64 (0.46 – 0.89)	0.009
> 1	Ref			
Palliative care (SPC)				
Yes	0.48 (0.34 – 0.69)	< .0001	0.41 (0.27 – 0.63) Ref	< 0.0001
No	Ref			
Nursing home resident				
Yes	0.86 (0.58 – 1.28)	0.46	0.46 (0.28 – 0.76)	0.002
No	Ref		Ref	

*OR* odds ratio, *SPC* specialized palliative care, *CCI* Charlson comorbidity index, *CI* confidence interval MOSAIC groups = socioeconomic entities based on small geographical areas, Mosaic group 1 corresponding to the most affluent one

In a separate multivariable analysis where nursing home residents were excluded, lower CCI indices were still associated with fewer ER visits and the association was somewhat strengthened for those receiving SPC with an OR 0.25 (0.15–0.42), *p* < 0.0001 (data not shown in Tables). Neither sex, age nor socio-economic Mosaic groups were associated with ER visits in multivariable analyses.

### Hospitalizations during the last month of life

Acute hospitalizations were needed in 33% of the patients during their last month of life: in 30% and 44% for those with and without SPC, respectively (*p* = 0.0006) (Table 3). In the logistic regression models, the outcomes were in essence similar with the findings as regards ER visits, except for an inverse association of female sex and hospitalizations (Table 5). When nursing home residents were removed from the complete multivariable analysis the association between receipt of SPC and risk of acute hospitalization was further strengthened with an OR of 0.24 (0.14–0.40), *p* < 0.0001, whereas the association of CCI and hospitalization became non-significant with OR of 0.73 (0.51 – 1.04), *p* = 0.08 (data not shown in Tables).

**Table 5:** Hospitalizations among brain tumor patients during the last month of life

Variable	Univariable analysis		Multivariable analysis	
	OR (95% CI)	*p*-value	OR (95% CI)	*p*-value
Sex				
Women	0.72 (0.53 – 0.98)	0.04	0.72 (0.53 – 0.99)	0.04
Men	Ref		Ref	
Socio-economic status				
Mosaic group 1	0.78 (0.53 – 1.15)	0.21	0.84 (0.56 – 1.26)	0.40
Mosaic group 2	0.78 (0.55 – 1.13)	0.19	0.85 (0.58 – 1.24)	0.40
Mosaic group 3	Ref		Ref	
Age groups				
18 – 64 years	0.92 (0.47 – 1.80)	0.80	1.05 (0.51 – 2.16)	0.89
65 – 74 years	0.84 (0.42 – 1.66)	0.62	0.95 (0.46 – 1.96)	0.89
75 – 84 years	1.04 (0.51 – 2.10)	0.92	1.12 (0.54 – 2.34)	0.76
85 years or older	Ref		Ref	
Comorbidity (CC1)				
0 – 1	0.60 (0.44 – 0.82)	0.001	0.65 (0.47 – 0.89)	0.007
> 1	Ref		Ref	
Palliative care (SPC)				
Yes	0.55 (0.39 – 0.78)	0.0007	0.45 (0.30 – 0.68)	0.0001
No	Ref		Ref	
Nursing home resident				
Yes	0.84 (0.57 – 1.22)	0.35	0.54 (0.34 – 0.85)	0.008
No	Ref		Ref	

*OR* odds ratio, *CI* confidence interval, *CCI* Charlson comorbidity index, *SPC* specialized palliative care; MOSAIC groups = socioeconomic entities based on small geographical areas, Mosaic group 1 corresponding to the most affluent one

### Acute hospitals as place of death

The characteristics of hospital deaths were similar to those of unplanned ER visits: receipt of SPC, being a nursing home resident or having a low CCI were all inversely associated with hospital deaths (Table 6). The difference between brain tumor patients receiving SPC and those who were not was striking: whereas 46/176 (26%) of patients without SPC died in acute hospitals, the corresponding figure was 14/604 (2%) for those receiving SPC (*p* < 0.0001) (Table 3). Neither sex, socio-economic Mosaic groups, nor age groups were associated with hospital deaths in univariable or multivariable models (Table 6). This was not changed in a separate multivariable analysis where nursing home residents were excluded (data not shown in Tables).

**Table 6:** Death in an acute hospital among patients with malignant brain tumors

Variable	Univariable analysis		Multivariable analysis	
	OR (95% CI)	*p*-value	OR (95% CI)	*p*-value
Sex				
Women	0.62 (0.35 – 1.08)	0.09	0.56 (0.28 – 1.12)	0.10
Men	Ref		Ref	
Socio-economic status				
Mosaic group 1	0.71 (0.37 – 1.36)	0.30	1.11 (0.49 – 2.49)	0.81
Mosaic group 2	0.56 (0.30 – 1.06)	0.07	0.78 (0.35 – 1.70)	0.52
Mosaic group 3	Ref		Ref	
Age groups				
18 – 64 years	1.30 (0.38 – 4.46)	0.68	2.53 (0.57 – 11.12)	0.22
65 – 74 years	0.99 (0.28 – 3.55)	0.99	1.80 (0.41 – 8.00)	0.44
75 – 84 years	0.95 (0.25 – 3.48)	0.95	1.16 (0.25 – 5.39)	0.85
85 years or older	Ref		Ref	
Comorbidity (CC1)				
0 – 1	0.32 (0.18 – 0.58)	0.0002	0.35 (0.17 – 0.73)	0.005
> 1	Ref		Ref	
Palliative care (SPC)				
Yes	0.07 (0.04 – 0.13)		0.03 (0.01 – 0.06)	
No	Ref	< .0001	Ref	< 0.0001
Nursing home resident				
Yes	0.49 (0.22 – 1.10)	0.08	0.08 (0.03 – 0.21)	< 0.0001
No	Ref		Ref	

*OR* odds ratio, *CI* confidence interval, *CCI* Charlson comorbidity index, *SPC* specialized palliative care, MOSAIC groups = socioeconomic entities based on small geographical areas, Mosaic group 1 corresponding to the most affluent one

## Discussion

The present study investigated factors associated with acute healthcare utilization in the last moth of life among brain tumor patients in the Stockholm area, the most populated health care region in Sweden (2.3 million inhabitants). Receipt of SPC in the last three months of life was equal in relation to sex and SES, as measured by Mosaic groups, whereas it was significantly higher for younger patients with less comorbidity and lower for nursing home residents. Unplanned ER visits and hospitalizations during the last month of life, as well as acute hospitals as a place of death were especially reduced in patients receiving SPC but also in patients who were nursing home residents. In contrast, neither SES nor age was associated with acute care in multivariable logistic models.

Inequality in cancer care, especially expensive treatments, has previously been described in relation to sex, age and SES, including educational level and marital status [28–30]. Moreover, cancer patients with lower SES have worse survival prospects [31]. The few published studies investigating equality of care for brain tumor patients point in the same direction [32–34]. To counteract this, equality in health and care is an expressed goal for the ongoing Swedish Cancer Plan, which is based on governmental guidelines and executed by the Regional Cancer Centres in Sweden [35]. Considering this, our finding that sex, when controlled for other variables, was not statistically associated and SES was only marginally associated with SPC is encouraging. The finding of an inverse association of age and SPC is in line with previous studies [36, 37].

Based on their meta-analysis, Henson et al. argue that unplanned ER visits as well as hospitalizations during the last month of life are associated with less optimal palliative care [12], findings that that are corroborated by others, in large studies comprising cancer and non-cancer patients [38, 39]. This is also in agreement with our previously published findings for cancer patients in Sweden [14]. Therefore, it is encouraging to find that the 77% of brain tumor patients who were receiving SPC were less likely to use acute care in the last month of life. In their meta-analysis with pooled data from several countries, the OR for unplanned emergency visits for those admitted to SPC was 0.43, well in line with the OR found in our study which was similar, OR 0.41. Henson, however, also reported that, men in contrast to women, as well as younger patients were more likely to visit the ER [12]. In our data, women were slightly less likely to be hospitalized in EOL, but sex was not associated with unplanned ER visits or hospital deaths, when controlling for relevant variables such as access to SPC, comorbidity and nursing home residency. Other authors have concluded that a higher Charlson Comorbidity index is associated with higher ER attendance, well in line with our studies [40].

Notably, receipt of SPC was more pronounced in the last 6 months and particular in the last 3 months in our study. Hence, despite typically suffering from complex and cumbersome symptoms, brain tumor patients were referred relatively late to SPC. From a clinical perspective, this could reflect the frequent observation that a decline in cognitive function and communication capabilities often tend to occur prior to severe physical deterioration. Nevertheless, it is conceivable that brain tumor patients and their families would benefit from earlier admission to SPC.

Previous studies have found that a majority would prefer to die at home, provided that adequate home care is guaranteed [41–44] although this may not be true for all patients [45, 46]. This is also corroborated in a Swedish study on patients in palliative home care, where 75% of those who had expressed a preferred place of death did wish to die at home, whereas 25% had a preference for dying in a palliative care ward [42].

In our material, 21% or the patients were nursing home residents and should be discussed separately from the whole group, as residents in Swedish nursing homes have high needs of assistance with activities of daily living (ADL), either due to several debilitating illnesses or due to cognitive failure [47]. In fact, a person is only referred to a nursing home after a municipal needs-assessor’s decision, and only when 6–8 scheduled visits per day by home-help services, including nightly visits, are insufficient to support a person with ADL in their own home. Those residing in nursing homes were, therefore, older and many of them were also likely to suffer from cognitive failure. When being a resident, efforts are made to provide adequate care in the nursing home and transfers to hospitals are avoided, when possible. This attitude is also corroborated by our previous finding of better quality of EOL cancer care in nursing homes as compared to acute hospitals [14, 29]. This was reflected in our findings in brain tumor patients of fewer of ER visits, hospitalizations, and hospital deaths for nursing home residents. For this reason, we performed separate multiple logistic regression analyses, to see whether analyses, where the nursing home residents were excluded, would result in other figures. When excluding nursing home residents, our main finding remained and was even strengthened: Receiving SPC was strongly associated with reduced use of acute hospital services. This may be relevant for several reasons: in addition to offloading the burden on acute hospitals, reduced acute care utilization in end-of-life may have beneficial effects on the quality of dying [12, 14, 48]. Notably, when patients are allowed to rate their preferred place of death, hospitals are ranked low [45]. Hence, our data with only 2% of hospital deaths among brain tumor patients admitted to SPC indicate a high quality of the palliative care. This is further corroborated by a Swedish study by Ozanne et al., who found that brain tumor patients in general receive good symptom control during their last week of life as regards pain, dyspnea, rattles and anxiety [49]. However, palliative home care services are highly dependent on informal family caregivers, as brain tumor patients often need extensive help with ADL for a long period of their disease trajectory, which is a huge commitment for the family [7, 50]. Therefore, support to the family is of great importance, also to relieve their own stress and anxiety [4, 5, 9].

### Strengths and limitations

A strength of this study was the use of a population-based cohort of brain tumor patients, with very few missing values, as reporting to the council´s administrative databases is mandatory and a basis for the economic compensation to the clinics.

The study has several limitations. First, we did not have access to the data from the National Cause of Death Register, hence, we identified the study subjects based on C71 as main diagnosis during the last year of life. We could therefore not be completely certain that the patients died from his/her brain tumor as opposed to with a malignant brain tumor but for some other reason. Second, the observational study design has inherent problems, e.g. we cannot assume that patients cared for in SPC have similar symptoms and care needs as patients who are nursing home residents (confounding by indication). Further related to the study design, since receipt of SPC was measured in the last 3 months of life it is possible that some patients may have received SPC after an acute care visit in the last month of life. This emphasizes the inability of our study to infer any conclusions on temporal or cause-effect relationships. Third, we used a decedent cohort, and it is possible that, for some patients death may have been unforeseen rendering both a lack of SPC as well as acute hospitalization ‘appropriate’ at the time. Moreover, inherent to the study design, we lacked both information on patient preferences with regards to desired place of death as well as knowledge on individual attitudes towards palliative care, i.e., some patients may have declined SPC and preferred to seek acute medical care upon deterioration. Forth, in order to estimate SES, we used MOSAIC which is a broad measure of SES that includes classical variables such as income and education but also additional variables such as lifestyle, cultural aspects, and living arrangement. MOSAIC groups may therefore be less specific than traditional measures of SES.

As regards generalizability, different models of palliative care services as well as models of financing must be taken into account. The data in this study are from the Stockholm region (2.3 million inhabitants) with well-developed SPC services, especially in the form of specialized palliative home care, and all health care is tax financed. Other countries might have different palliative care models, as well as models for financing, which might affect the results as regards the degree of hospitalization.

## Conclusion

SPC in the last three months of life was associated with lower acute health care utilization and improved the chances of avoiding death in acute hospitals. Brain tumor patients with multiple comorbidities were more likely to require acute care whereas nursing home residency was a protective factor.

## Data Availability

The datasets generated, used and analyzed during the current study available from the corresponding author on reasonable request.

## Abbreviations

EOL: End-of-life
OR: Odds ratio
SES: Socioeconomic status
ER: Emergency room

## References

[1] Wu A, Ruiz Colón G, Aslakson R, Pollom E, Patel CB: Palliative Care Service Utilization and Advance Care Planning for Adult Glioblastoma Patients: A Systematic Review. Cancers (Basel) 2021, 13(12). 10.3390/cancers13122867 PMC8228109 34201260

[2] Gould J (2018). Breaking down the epidemiology of brain cancer. Nature.

[3] Tumörer i hjärna och ryggmärg. Nationellt vårdprogram. (Tumors of brain and spinal cord. National guidelines) [https://kunskapsbanken.cancercentrum.se/diagnoser/hjarna/]

[4] Pace A, Dirven L, Koekkoek JAF, Golla H, Fleming J, Rudà R, Marosi C, Le Rhun E, Grant R, Oliver K (2017). European Association for Neuro-Oncology (EANO) guidelines for palliative care in adults with glioma. Lancet Oncol.

[5] Kluger BM, Ney DE, Bagley SJ, Mohile N, Taylor LP, Walbert T, Jones CA (2020). Top Ten Tips Palliative Care Clinicians Should Know When Caring for Patients with Brain Cancer. J Palliat Med.

[6] Siegel C, Armstrong TS (2018). Nursing Guide to Management of Major Symptoms in Patients with Malignant Glioma. Semin Oncol Nurs.

[7] Giammalva GR, Iacopino DG, Azzarello G, Gaggiotti C, Graziano F, Gulì C, Pino MA, Maugeri R: End-of-Life Care in High-Grade Glioma Patients. The Palliative and Supportive Perspective. Brain Sci 2018, 8(7). 10.3390/brainsci8070125 PMC6071221 29966347

[8] Collins A, Lethborg C, Brand C, Gold M, Moore G, Sundararajan V, Murphy M, Philip J (2014). The challenges and suffering of caring for people with primary malignant glioma: qualitative perspectives on improving current supportive and palliative care practices. BMJ Support Palliat Care.

[9] Moore G, Collins A, Brand C, Gold M, Lethborg C, Murphy M, Sundararajan V, Philip J (2013). Palliative and supportive care needs of patients with high-grade glioma and their carers: a systematic review of qualitative literature. Patient Educ Couns.

[10] Nordström M, Strang P (2018). High Degree of Satisfaction With the Support Given by Multidisciplinary Palliative Home Care Teams in the County of Stockholm. J Palliat Care.

[11] Ferrell BR, Temel JS, Temin S, Alesi ER, Balboni TA, Basch EM, Firn JI, Paice JA, Peppercorn JM, Phillips T (2017). Integration of Palliative Care Into Standard Oncology Care: American Society of Clinical Oncology Clinical Practice Guideline Update. J Clin Oncol.

[12] Henson LA, Gao W, Higginson IJ, Smith M, Davies JM, Ellis-Smith C, Daveson BA (2015). Emergency department attendance by patients with cancer in their last month of life: a systematic review and meta-analysis. J Clin Oncol.

[13] Wright AA, Keating NL, Ayanian JZ, Chrischilles EA, Kahn KL, Ritchie CS, Weeks JC, Earle CC, Landrum MB (2016). Family Perspectives on Aggressive Cancer Care Near the End of Life. JAMA.

[14] Elmstedt S, Mogensen H, Hallmans DE, Tavelin B, Lundström S, Lindskog M (2019). Cancer patients hospitalised in the last week of life risk insufficient care quality - a population-based study from the Swedish Register of Palliative Care. Acta Oncol.

[15] Cavalli-Bjorkman N, Glimelius B, Strang P: Equal cancer treatment regardless of education level and family support? A qualitative study of oncologists' decision-making. BMJ Open 2012, 2(4). 10.1136/bmjopen-2012-001248 PMC3432847 22923630

[16] Cavalli-Bjorkman N (2014). Implications of patients' socioeconomic status - what oncologists should be aware of. Acta Oncol.

[17] Fürst P, Strang P, Hedman C, Schultz T: Advanced cancer and concomitant dementia: access to specialized palliative care, emergency room, hospital care, and place of death. Acta Oncol 2022:1–7. 10.1080/0284186X.2022.2062681 35411838

[18] D'Angelo D, Di Nitto M, Giannarelli D, Croci I, Latina R, Marchetti A, Magnani C, Mastroianni C, Piredda M, Artico M (2020). Inequity in palliative care service full utilisation among patients with advanced cancer: a retrospective Cohort study. Acta Oncol.

[19] Nilsson J, Holgersson G, Ullenhag G, Holmgren M, Axelsson B, Carlsson T, Bergqvist M, Bergström S (2021). Socioeconomy as a prognostic factor for location of death in Swedish palliative cancer patients. BMC Palliat Care.

[20] Diamond EL, Panageas KS, Dallara A, Pollock A, Applebaum AJ, Carver AC, Pentsova E, DeAngelis LM, Prigerson HG (2017). Frequency and Predictors of Acute Hospitalization Before Death in Patients With Glioblastoma. J Pain Symptom Manage.

[21] Alturki A, Gagnon B, Petrecca K, Scott SC, Nadeau L, Mayo N (2014). Patterns of care at end of life for people with primary intracranial tumors: lessons learned. J Neurooncol.

[22] Sundararajan V, Bohensky MA, Moore G, Brand CA, Lethborg C, Gold M, Murphy MA, Collins A, Philip J (2014). Mapping the patterns of care, the receipt of palliative care and the site of death for patients with malignant glioma. J Neurooncol.

[23] Vandenbroucke JP, von Elm E, Altman DG, Gotzsche PC, Mulrow CD, Pocock SJ, Poole C, Schlesselman JJ, Egger M, Initiative S (2007). Strengthening the Reporting of Observational Studies in Epidemiology (STROBE): explanation and elaboration. Epidemiology.

[24] Dahlén E, Komen J, Jonsson EW, Almqvist C, Kull I, Wettermark B (2019). Eliminated patient fee and changes in dispensing patterns of asthma medication in children-An interrupted time series analysis. Basic Clin Pharmacol Toxicol.

[25] InsightOne: Experia MIS Mosaic^TM^ Sweden. In*.*; 2015.

[26] Strang  P, Furst  P, Schultz  T (2020). Excess deaths from COVID-19 correlate with age and socio-economic status. A database study in the Stockholm region. Ups J Med Sci.

[27] Charlson ME, Pompei P, Ales KL, MacKenzie CR (1987). A new method of classifying prognostic comorbidity in longitudinal studies: development and validation. J Chronic Dis.

[28] Cavalli-Bjorkman N, Lambe M, Eaker S, Sandin F, Glimelius B (2011). Differences according to educational level in the management and survival of colorectal cancer in Sweden. Eur J Cancer.

[29] Cavalli-Bjorkman N, Qvortrup C, Sebjornsen S, Pfeiffer P, Wentzel-Larsen T, Glimelius B, Sorbye H (2012). Lower treatment intensity and poorer survival in metastatic colorectal cancer patients who live alone. Br J Cancer.

[30] Nilssen Y, Eriksen MT, Guren MG, Møller B (2021). Factors associated with emergency-onset diagnosis, time to treatment and type of treatment in colorectal cancer patients in Norway. BMC Cancer.

[31] Afshar N, English DR, Milne RL (2021). Factors Explaining Socio-Economic Inequalities in Cancer Survival: A Systematic Review. Cancer Control.

[32] Cote DJ, Ostrom QT, Gittleman H, Duncan KR, CreveCoeur TS, Kruchko C, Smith TR, Stampfer MJ, Barnholtz-Sloan JS (2019). Glioma incidence and survival variations by county-level socioeconomic measures. Cancer.

[33] Deb S, Pendharkar AV, Schoen MK, Altekruse S, Ratliff J, Desai A (2017). The effect of socioeconomic status on gross total resection, radiation therapy and overall survival in patients with gliomas. J Neurooncol.

[34] Ryan CS, Juhn YJ, Kaur H, Wi CI, Ryu E, King KS, Lachance DH (2020). Long-term incidence of glioma in Olmsted County, Minnesota, and disparities in postglioma survival rate: a population-based study. Neurooncol Pract.

[35] Regional Cancer Plan (Swe: Regional cancerplan för Stockholm-Gotland) [https://cancercentrum.se/stockholm-gotland/om-oss/strategisk-utvecklingsplan/cancerplan-2020-2023/]

[36] Strang P, Furst P, Hedman C, Bergqvist J, Adlitzer H, Schultz T (2021). Chronic obstructive pulmonary disease and lung cancer: access to palliative care, emergency room visits and hospital deaths. BMC Pulm Med.

[37] Lindskog M, Tavelin B, Lundström S (2015). Old age as risk indicator for poor end-of-life care quality - a population-based study of cancer deaths from the Swedish Register of Palliative Care. Eur J Cancer.

[38] Bone AE, Evans CJ, Etkind SN, Sleeman KE, Gomes B, Aldridge M, Keep J, Verne J, Higginson IJ (2019). Factors associated with older people's emergency department attendance towards the end of life: a systematic review. Eur J Public Health.

[39] Lin CP, Tsay MS, Chang YH, Chen HC, Wang CY, Chuang YS, Wu CY: A Comparison of the Survival, Place of Death, and Medical Utilization of Terminal Patients Receiving Hospital-Based and Community-Based Palliative Home Care: A Retrospective and Propensity Score Matching Cohort Study. Int J Environ Res Public Health 2021, 18(14). 10.3390/ijerph18147272 PMC8307712 34299722

[40] Lamba N, Catalano PJ, Whitehouse C, Martin KL, Mendu ML, Haas-Kogan DA, Wen PY, Aizer AA (2021). Emergency department visits and inpatient hospitalizations among older patients with brain metastases: a dual population- and institution-level analysis. Neurooncol Pract.

[41] Cai J, Zhang L, Guerriere D, Coyte PC (2020). Congruence between Preferred and Actual Place of Death for Those in Receipt of Home-Based Palliative Care. J Palliat Med.

[42] Rasch-Westin M, Helde-Frankling M, Björkhem-Bergman L: Death at home: predictive factors in a medical home care unit. BMJ Support Palliat Care 2019. 10.1136/bmjspcare-2019-001932 31537581

[43] Arnold E, Finucane AM, Oxenham D (2015). Preferred place of death for patients referred to a specialist palliative care service. BMJ Support Palliat Care.

[44] Rainsford S, MacLeod RD, Glasgow NJ (2016). Place of death in rural palliative care: A systematic review. Palliat Med.

[45] Higginson IJ, Daveson BA, Morrison RS, Yi D, Meier D, Smith M, Ryan K, McQuillan R, Johnston BM, Normand C (2017). Social and clinical determinants of preferences and their achievement at the end of life: prospective cohort study of older adults receiving palliative care in three countries. BMC Geriatr.

[46] Pollock K (2015). Is home always the best and preferred place of death?. BMJ.

[47] Bjork S, Juthberg C, Lindkvist M, Wimo A, Sandman PO, Winblad B, Edvardsson D (2016). Exploring the prevalence and variance of cognitive impairment, pain, neuropsychiatric symptoms and ADL dependency among persons living in nursing homes; a cross-sectional study. BMC Geriatr.

[48] Sizoo EM, Taphoorn MJ, Uitdehaag B, Heimans JJ, Deliens L, Reijneveld JC, Pasman HR (2013). The end-of-life phase of high-grade glioma patients: dying with dignity?. Oncologist.

[49] Ozanne A, Sawatzky R, Håkanson C, Alvariza A, Fürst CJ, Årestedt K, Öhlén J (2019). Symptom relief during last week of life in neurological diseases. Brain Behav.

[50] Philip J, Collins A, Brand C, Sundararajan V, Lethborg C, Gold M, Lau R, Moore G, Murphy M (2018). A proposed framework of supportive and palliative care for people with high-grade glioma. Neuro Oncol.

